# Applications of Polymeric Composites in Bone Tissue Engineering and Jawbone Regeneration

**DOI:** 10.3390/polym13193429

**Published:** 2021-10-06

**Authors:** Oscar Fraile-Martínez, Cielo García-Montero, Alejandro Coca, Miguel Angel Álvarez-Mon, Jorge Monserrat, Ana M. Gómez-Lahoz, Santiago Coca, Melchor Álvarez-Mon, Julio Acero, Julia Bujan, Natalio García-Honduvilla, Ángel Asúnsolo, Miguel A. Ortega

**Affiliations:** 1 Department of Medicine and Medical Specialities, University of Alcalá, 28801 Alcalá de Henares, Spain; oscarfra.7@hotmail.com (O.F.-M.); cielo.gmontero@gmail.com (C.G.-M.); coca125@hotmail.com (A.C.); maalvarezdemon@icloud.com (M.A.Á.-M.); jorge.monserrat@uah.es (J.M.); alahoz1199@gmail.com (A.M.G.-L.); s.coca@uah.es (S.C.); mademons@gmail.com (M.Á.-M.); mjulia.bujan@uah.es (J.B.); natalio.garcia@uah.es (N.G.-H.); miguel.angel.ortega92@gmail.com (M.A.O.); 2 Ramón y Cajal Institute of Sanitary Research (IRYCIS), 28034 Madrid, Spain; 3 Immune System Diseases-Rheumatology, Oncology Service and Internal Medicine, University Hospital Príncipe de Asturias (CIBEREHD), 28806 Alcalá de Henares, Spain; 4 Department of Surgery, Medical and Social Sciences, Faculty of Medicine and Health Sciences, University of Alcalá, 28801 Alcalá de Henares, Spain; julio.acero@uah.es; 5 Department of Oral and Maxillofacial Surgery, Ramon y Cajal University Hospital, University of Alcalá, 28034 Madrid, Spain; 6 Cancer Registry and Pathology Department, Hospital Universitario Principe de Asturias, 28806 Alcalá de Henares, Spain

**Keywords:** polymeric composites, bone tissue engineering, hydroxyapatite/collagen, jawbone, regenerative medicine

## Abstract

Polymer-based composites are a group of biomaterials that exert synergic and combined activity. There are multiple reported uses of these composites in multiple biomedical areas, such as drug carriers, in wound dressings, and, more prominently, in tissue engineering and regenerative medicine. Bone grafting is a promising field in the use of polymeric composites, as this is the second most frequently transplanted organ in the United States. Advances in novel biomaterials, such as polymeric composites, will undoubtedly be of great aid in bone tissue engineering and regeneration. In this paper, a general view of bone structure and polymeric composites will be given, discussing the potential role of these components in bone tissue. Moreover, the most relevant jawbone and maxillofacial applications of polymeric composites will be revised in this article, collecting the main knowledge about this topic and emphasizing the need of further clinical studies in humans.

## 1. Introduction

Bone tissue is a dynamic structure with great adaptative properties to the signals of the environment. Indeed, bone tissue is continuously being synthesized and reabsorbed in a process controlled by many local and systemic molecules, therefore regulating bone homeostasis. Similarly, mechanical forces can reinforce the proper structure of the bone, whereas the exposure to acute or chronic stressors could lead to bone injuries and fractures, which may activate the process of bone repair [1]. This process is achieved in the following three simple stages: the initial inflammatory response, bone formation, and bone remodeling, with a plethora of cells and molecules participating in these phases [2,3]. Despite bone regeneration after fractures generally being completed without the formation of any scar tissue, there are some other fractures where bone repair is impaired and requires some medical interventions. This is the case of larger orthopedic and oral/maxillofacial surgeries, generally due to infections, tumor resections, traumas, skeletal abnormalities or other conditions, such as osteoporosis or avascular necrosis, in which a large bone repair is required, even beyond the self-regeneration capacity [4,5]. Bone grafting is a common procedure used in orthopedic and maxillofacial interventions, being the second most frequently transplanted tissue in the United States [6]. Autogenous cancellous bone graft is considered the gold standard of bone grafting because of its osteogenic, osteoinductive and osteoconductive properties, although it is associated with increased host morbidity [7]. Osteoinduction refers to the process by which osteogenesis is induced. An osteoconductive surface is a structure that permits new bone to be formed, and osseointegration is the fixation of the graft in the bone [8]. Allograft bones present some advantages, such as their abundance, convenience, and lack of procurement-related patient morbidity [6]. However, there are some issues concerning this option, as it could be associated with a risk of infection and graft rejection. Indeed, about 50% of patients report a sensitized immune system after receiving an allogeneic bone graft, with unknown local and systemic consequences [9], also showing decreased osteogenic and osteoinductive characteristics [10]. Thereby, bone graft substitutes have been receiving growing attention as a potential alternative to the previous strategies. In this sense, there are growth factor-based, cell-based, ceramic-based, polymer-based or miscellaneous bone graft substitutes that may be used alone or in combination with other materials [11]. Polymeric composites are combinations of two or more components that exert their action jointly. There is much research supporting the numerous applications of polymer composites in different biomedical fields, including bone and maxillofacial regeneration [12,13,14]. The present review aims to collect the main knowledge and applications of polymer composites, their structure, and the rationale for their use in bone engineering tissue, with special regard to jaw regeneration.

## 2. Bone Cytoarchitecture and Remodeling

**Figure 1:**
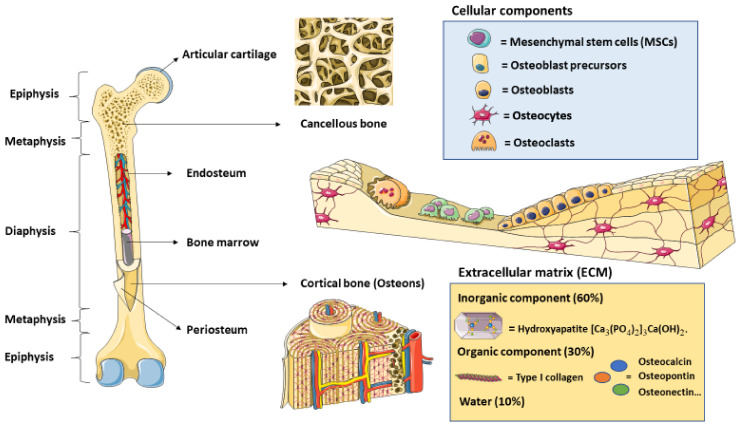
An integrative perspective of bone anatomy, histology and cellular/molecular components. In this picture, the main anatomical structures of the long bones may be distinguished, including the epiphysis, metaphysis and diaphysis, together with the main bone layers. These are, from outer to inner, periosteum, cortical bone (in diaphysis) or cancellous bone (in the epiphysis and metaphysis), endosteum and bone marrow. The histological structure is also reviewed, with special emphasis on the cellular components, composed of mesenchymal stem cells, osteoblasts and their precursors, osteocytes and osteoclasts as well as the extracellular matrix, mainly formed by the inorganic element hydroxyapatite (60%) followed by the organic component (30%), prominently type I collagen although other proteins, such as osteonectin osteopontin or osteocalcin, must also be considered and water (10%).

Examining the potential applications of polymer composites in bone regeneration is crucial to understand bone cytoarchitecture and its behavior. Bones are the major components of the human skeleton, playing a critical role in locomotion and in the protection of vital organs, providing structural and functional support for hematopoiesis, as well as participating in mineral and acid–base balance homeostasis [15]. At the microscopic level, bone cells and extracellular matrix components must be distinguished. Osteoblasts are cells involved in the osteogenesis process, and they play a critical role in bone remodeling [16]. They are derived from mesenchymal stem cells (MSCs), which are multipotent cells that have been studied for some time in the field of regenerative medicine, including in bone repair and regeneration [17,18]. Conversely, osteoclasts are multinucleated cells that are derived from myeloid hematopoietic stem cells, which are responsible for bone resorption [19]. The balance between osteoblasts and osteoclasts is key for bone homeostasis, and an abnormal activity of these two populations may be responsible for disease conditions such as osteoporosis [20]. Osteocytes, which derive from osteoblasts, are the major cell population in bone. They are essential for maintaining bone morphology and act as mechanosensors, thereby orchestrating bone remodeling and an adaptative response to the environment [21]. Regarding the extracellular components, bone is 10% water, 30% organic, and 60% inorganic. The organic components are mainly represented by type I collagen, representing between 80 and 90% of the total, although non-collagenous proteins (osteocalcin and osteonectin), glycoproteins (osteopontin), and proteoglycans are equally important in this tissue [22]. The inorganic component, also named the mineralized matrix, is primarily composed of hydroxyapatite (HA) crystals [Ca_3_(PO4)_2_]_3_Ca (OH)_2_. By volume, 40% of bone corresponds to the inorganic component (HA), 25% to water, and 35% to the organic component. This inorganic component is critical as a mineral repository for calcium and phosphate, also containing impurities that reduce the crystallinity of the bone mineral, which is important for mineral homeostasis and bone adaptation [23]. From a histological perspective, bone is composed of different parts. Periosteum is the most external structure present in almost all bones. It is composed of an outer fibrous layer, mainly characterized by low cell populations and a greater ECM. It could be subdivided into a highly vascularized superficial portion, mainly composed of collagen and a few elastic fibers, and a deep portion with many elastic fibers and collagen. Conversely, the inner cambium layer of the periosteum is highly cellular, with many MSCs, differentiated osteogenic progenitor cells, osteoblasts, fibroblasts, and a poor ECM [24]. More internally, cortical (compact) bone and cancellous (trabecular) bone could be distinguished. The first represents approximately 80% of the total bone in the body, being notably stronger than the second one. Frequently, cortical bone is found in the shaft of long bones, also known as diaphysis, protecting the medullary cavity. In more detail, cortical bone is composed of osteons, which represent the structural and functional unit [25]. In contrast, cancellous bone is characterized by high porosity, which gives this structure significant mechanobiological properties [26]. Indeed, cancellous bone responds eight times faster to changes in load, and has ten times the surface/volume ratio of cortical bone [27]. In addition, cancellous bone is detected in the end of long bones, both at the metaphysis (below the growth plates) and the epiphysis (above the growth plates), where there is no medullary cavity [28]. Endosteum is an inner membrane that is notably thinner than periosteum, revesting the bone marrow cavity, the osteons, and the trabecula near the developing part of the bone. It is an essential structure that is formed by osteoprogenitor cells and type III collagen fibers (reticular fibers) [29]. Although bone marrow is considered as part of the hematological rather than osseus tissue, it could also play important roles in the bone repair and regeneration process, due to its abundance of MSCs [30]. Having integrative knowledge of bone cells and ECM components, the structures formed, and the function of each part will provide many benefits in the field of bone tissue engineering (Figure 1). However, previous works have noticed many difficulties in this field, as it is not as simple as combining different cell types with some scaffolds, growth factors, and other components, and waiting for a complete regeneration. It is undeniable that bone tissue engineering is still in its infancy, and many efforts are required to find the most suitable strategies while individualizing for each case [31]. In this context, reviewing the different studies of polymer composites in bone regenerative medicine may aid in understanding the important roles that this approach may have, as well as aiding the establishment of future studies on these techniques.

## 3. Polymeric Composites: Concept, Technology and Biomedical Applications

**Figure 2:**
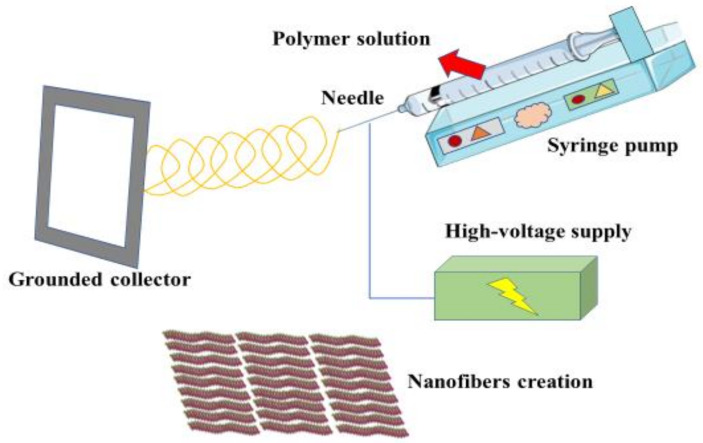
A general picture of the electrospinning method. As shown, the polymer solution is forced through a needle by using the syringe pump. The needle receives a high voltage supply, injecting a charge of a particular polarity into the polymer solution. This creates a repulsion of similar changes that eventually lead to the formation of nanofibers, which are deposited onto the grounded collector.

As previously defined, polymeric composites are multi-phase materials with synergic mechanical properties that are not achieved from each component alone. Polymeric composites are composed of at least a matrix phase, which is more ductile and less hard, and a dispersed (reinforcing) phase. Polymeric composites have the following characteristics: (1) they usually consist of two or more physically unique and mechanically separable materials, (2) they are made in such a way to achieve a uniform and controlled dispersion of their constituents, and (3) they exert superior mechanical properties, which are occasionally different from their integrators [32]. According to the matrix phase, composites could be divided into metal matrix composites, ceramic matrix composites, and polymer matrix composites (PMCs). Simultaneously, the reinforcing fibers could be distinguished among particulate, laminate and fibrous composites. The last one, in turn, could be subdivided into synthetic fibers and natural fibers. Natural fibers can be classified if they are embedded in a non-degradable or biodegradable matrix. The latter are referred to as green composites. Eventually, green composites could be defined as textile or hybrid composites, which are those composed of two or more types of fibers [13,32]. PMCs are broadly extended and categorized due to their simple fabrication process, low cost, and availability. Some polymers with biomedical applications are polyethylene (PE), polyurethane (PU) polymethylmethacrylate (PMMA), silicone rubber (SR), poly(lactic acid) (PLA), and poly(glycolic acid) (PGA), and in a few polymeric composite biomaterials we can distinguish between HA/PE, carbon fiber/ultra-high-molecular-weight polyethylene (CF/UHMWPE), or carbon fiber/epoxy (CF/epoxy) [33]. Different strategies are described in the fabrication of polymer composites, including electrospinning, solution mixing, melt extrusion, latex technology, and in situ methods [13]. The first method, electrospinning, is an effective one-step technique to construct polymeric nanofibers and their composites, with a diameter between nanometers and micrometers, frequently reinforced with nanocarbons, such as carbon nanotubes, graphenes, nanodiamonds, nanodots, and many others [34]. The different electrospinning components are as follows: a high-voltage power source, an electrically conductive spinneret, a syringe pump, a grounded collector, and a polymer solution. As shown in Figure 2, the polymer solution is forced through a needle by using the syringe pump. The needle receives a high voltage supply, injecting a charge of a particular polarity into the polymer solution, creating a repulsion of similar changes that eventually lead to the formation of nanofibers, which are deposited onto the grounded collector [35]. This method reports some advantages, including its cost effectiveness, simplicity, and high production rates. Conversely, it also presents some difficulties regarding the fabrication and consistency of organic and inorganic nanofibers, as well as the greater costs in the production of large-diameter nanofibers [34]. Solution mixing consists of the dissolution of a polymer at a particular temperature in a solvent, and then a homogeneous distribution of fillers, such as montmorillonite clays, into the polymer solution [13]. Melt extrusion is an effective technique used in many pharmaceuticals applications, including the formation of pellets, tablets, granules, suppositories, implants, stents, transdermal systems, and ophthalmic inserts [36]. This method is based on the conversion of different raw materials with counter-rotating or co-rotating screw elements in an extruder, being submitted to high temperatures to melt and mix the components [37]. This technique is used for making fiber-reinforced compounds, although a thorough simulation model is needed before performing the synthesis of polymer composites by this method [38]. Latex technology is a unique and environmentally friendly process by which polymeric nano- and micro-composites may be formed [39]. The entire process is applicable to multiple fillers and polymers. This approach could be particularly useful for targeting some characteristics of the polymeric composite, such as the thermal conductivity [40]. Finally, the in situ approach consists of the mixture of the matrix material and metal ions, followed by exposure to the counterion (S2^−^, Se2^−^) in the form of gas or dissolved ions. Then, the composite may be cast as a film before or after the exposure to the counterion [41,42]. Recently, Shuai et al. [43] developed a core–shell-structured nanoparticle that was composed of zeolitic imidazolate framework-8 (ZIF-8) as the core and HA as the shell, constructed by the polydopamine (PDA)-induced in situ growth of HA on ZIF-8 nanoparticles. This procedure augmented the bioactivity of the HA, while it enhanced the mineralization ability of the scaffolds and promoted cell adhesion, proliferation, and differentiation. Similar results were obtained from Yang et al. [44], who synthesized a composite formed by HA, silver, and PLLA, following the same methodology, reporting excellent antimicrobial uses, bone regeneration, and bioactivity, without generating an inflammatory response. As shown, novel methodological approaches are arising to develop more effective polymer composites, maximizing their properties and extending their applications.

Polymer composites have a plethora of medical and non-medical uses, including those in aerospace, military, sportive and automobile applications. In the biomedical field, they are being studied as models of drug release, wound dressings, surgeries, odontology, and, as reviewed in this paper, in tissue engineering and regenerative medicine [45]. Many of the tissues in living organisms are composites, as they are made up of different constituents that, in their proportion, distribution, morphology, and properties, synergically determine the final functioning of the tissue and organs. Indeed, there is an increasing number of studies supporting the various applications of polymer composites in a wide variety of body structures, such as bone, muscle, cartilage, blood vessels, nerves, and heart valves, among others [46]. In the next section, we will summarize some of the most important polymers applicated to bone regenerative medicine.

## 4. Most Relevant Polymeric Composites in Bone Regeneration

The evolution of bone graft biomaterials included four different generations. The first-generation bone grafts were metals, with limited properties in their bioresorption and bioactivity, with repetitive surgeries needed to replace these materials. Later, second-generation biomaterials included bioactive ceramics and bioresorbable polymers, which showed, however, certain limitations that were ameliorated in third-generation bone grafts, using composites that combined both bioactive and bioresorbable properties, addressing the regeneration of living tissues at the molecular and cellular levels [47]. Now, we are in the era of fourth-generation biomaterials, also known as smart or biomimetic materials. They are polymer–ceramic composite scaffolds with osteogenic cells, growth factors, or bone morphogenetic proteins, either alone or in combination. The aim of these biomaterials is to emulate the behavior of the proper tissue, even by targeting its bioelectric properties [48,49,50].

As previously mentioned, 40% of the bone volume is the mineral material HA. The brittle character of this ceramic material does not allow it to be applied alone, but its synthetic production maintains naïve bioactive, biocompatible and osteoconductive properties to be used as part of scaffold composites [14]. HA is a resistant material with limitations associated with its flexibility. It is supplied by being combined with polymers such as collagen constituting polymer–ceramic composites [48]. Collagen/HA is a natural composite, with combined properties and great osteoinduction. Type I collagen is one of the main organic components of bone and the main organic component of the extracellular matrix, and provides adaptation to defect morphology at implant sites, due to the elastic and sticky features [51]. In fact, 1960s collagen was present in the development of biomaterials in the era of early tissue engineering. It was obtained from animal sources and was easily manufactured to create sponges, hydrogels, or fibers to attach to HA [52].

For its part, HA compression strength has already been evaluated in vivo two decades ago, showing similar behavior to natural cortical bone [53]. Compression testing for different degrees of porosity in hydroxyapatite porous scaffolds revealed that the microporous form provides better osteoconductivity and stress responses than non-microporous scaffolds, as well as better growth factor retention and drug carrier ability [54]. From this point, in the following years, nanotechnology and 3D printing have provided pivotal work to build nanostructured materials (nano-HA, nHA) that ease protein attachment and apatite synthesis, emulating bone construction [55]. For cranioplasty, nHA has been tested in vivo to check its response to functionalization with bone morphogenic protein-2 (BMP-2) and zoledronic acid, and carrying bioactive molecules [56]. In contrast, in the field of maxillofacial and dentoalveolar deficiencies, different collagen/HA ratios, and the controlled mineralization of nano-crystals in situ, have been tested in vitro and in vivo to check biocompatibility and biomimetic properties, corroborating the suitability of this natural composite for bone graft scaffolds [57]. New dental cements also include these nano-crystals now that they have a powerful ability to remineralize enamel lesions without affecting the sensitivity of teeth, even better than fluoride [58], and it has been proved that is not toxic as an oral care ingredient, showing cytocompatibility with gingival cells [59].

According to the literature, collagen/HA could be considered as the basis for building an ideal bone graft scaffold, respecting the natural composite and conferring great bioactivity. Despite the good biocompatibility, type I collagen is highly biodegradable and does not provide enough mechanical strength [52], although different forms of collagen barrier membranes have been tested in the resorption of bone regeneration (with differences from a few hours to 21 days) [60]. In most cases, however, this time degradation needs to be longer, the mechanical properties need to be enhanced, and other functions are desired to be supplied One example is the reinforcement of HA with CaO, which is then combined with collagen, showing potential as a bone graft with a longer time of post-operation bone regeneration [61]. Another approach is the design of multifunctional bone implants with gel silica plus Col/HA, for the treatment of diseases that require drug delivery besides implants [62]. For bone repair defects, some experiments have played with scales of nHA in 3D, with human-like collagen and cross-linked by diepoxyoctane, altogether denoting anti-biodegradation and great mechanical properties. The objective of these studies was to evaluate which level of crosslinking allowed better cytocompatibility and histocompatibility [63]. Furthermore, exploring the optimization by the addition of biomaterials derived from non-collagenous extracellular bone matrix proteins (e.g., osteonectin, fibronectin, vitronectin…) is also key to regulate biological processes in bone regeneration, such as growth factor activation, cell migration, proliferation, osteogenesis, or angiogenesis [64].

New approaches to obtain a multifunctional scaffold, both for bone regeneration and load bearing, have focused on the combination of gels and synthetic polymers. In vitro studies of microspheres of poly-lactic-co-glycolic acid (PLGA) with nHA combined with gelatin/nHA cryogel showed an empowered Young’s modulus and stress response. These parameters even improved with stem cell adhesion and proliferation, also accompanied by an adequate expression of osteogenic marker genes, which are key for bone regeneration [65]. For skull defects, the coating of PLGA/HA with Asp-Gly-Glu-Ala collagen also showed promising results in vivo, once again improving the scaffold base properties [66]. Playing with different shapes of composites at nanoscales was the case in other in vitro and in vivo studies for mandible defects. Insulin-loaded PLGA nanospheres were introduced into collagen/nHA scaffolds to verify the osteogenesis function of insulin when it is released from PLGA nanospheres, aiding bone marrow mesenchymal stem cell adhesion, proliferation, and differentiation [67].

**Figure 3:**
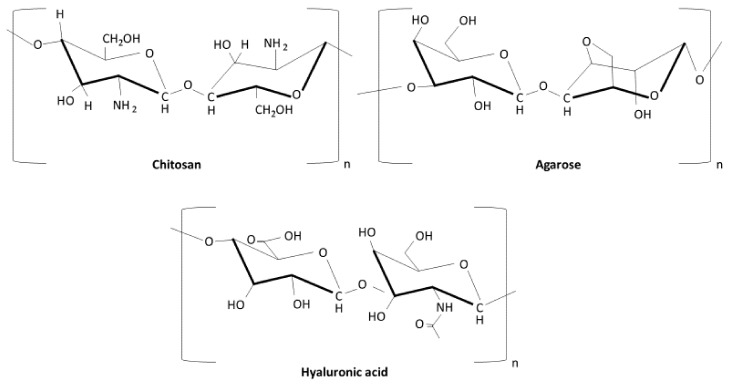
Chemical structures of the main polymer biomaterials.

Agarose and chitosan are materials that present certain advantages for bone tissue engineering, due to their similar hydrophilic behavior to the extracellular matrix of bone. Agarose (Figure 3) is a natural polymer of 1,3-linked-D-galactopyranose and 1,4-linked 3,6-anhydro-α-L-galactopyranose, which has demonstrated the following special characteristics for biomedical applications: thermo-reversible gelation behavior, and it has been shown to be a good material for controlled and localized drug delivery [68]. Moreover, agarose provides extra flexibility to the composite, making HA-based scaffolds easy to handle by surgeons [69]. Chitosan (Figure 3) is another natural polymer of β-(1-4)-linked d-glucosamine and N-acetyl-d-glucosamine, which is derived from chitin and is widely used in biomedical applications, thanks to its low allergenicity, biocompatibility, and biodegradability [70]. This material provides a good substrate to the composite, for the adhesion and proliferation of osteoblasts, and also matrix formation, and is able to mimic the shape and size of structural proteins of the bone matrix [71]. All in all, both polymers also provide a good medium for the delivery of growth factors in bone regeneration. The use of foaming agents in the production of microporous nanocomposite materials has been tested for the bone healing process, concretely, chitosan/agarose/nHA, which is based on the reinforcement of chitosan and agarose polysaccharides with nanoparticles of nHA [72]. These highly microporous composites are interesting for non-load-bearing implantation sites, considering that, although high porosity enables cell attachment and growth, the biomechanical strength is lower [73]. Hyaluronic acid (Figure 3) is another polymer that consists of β-1,4-D-glucuronic acid and β-1,3-N-acetylglucosamine units. It is produced either endogenously in the cells of the body or by microbial fermentation, and has been studied in different biomedical applications [74]. In the field of bone tissue engineering, hyaluronic acid has been tested in combination with HA and collagen, showing similar properties to HA and collagen alone, but higher cohesivity and greater biocompatibility [75]. Further, Chang et al. [76] evaluated the use of HA with beta-tricalcium phosphate (β-TCP) and hyaluronic acid in a rabbit model, reporting that the application of this component improved the osteoconductive properties and handling characteristics in clinical situations. Moreover, the incorporation of hyaluronic acid and its derivates into different composite scaffolds improves osteogenesis and mineralization, and they also serve as potential carriers of various osteoinductive products, thereby improving osseointegration [77]. Thus, the use of hyaluronic acid has been claimed to be a promising tool in bone regeneration. In the same manner, elastin-like polypeptide (ELP) also appears to be a relevant approach, improving the mechanical properties when combined with collagen [78], bone mineralization and regeneration [79], and as a delivery system for different components, such as the growth factor BMP-2 [80]. Better results could be accomplished when it was combined with collagen and bioglass scaffolds, as the synergic properties of each component may aid in the bone healing process [81].

The physicochemical properties and knowledge of the novel possibilities of the combination of materials keep rising. Silk fibroin (SF), produced by silkworms and spiders, has revolutionized tissue engineering as well. Its versatility resides in its tunable biodegradation and mechanical properties [82]. Its elasticity and flexibility also aid to attenuate HA brittleness [83]. Additionally, injectable SF/gelatin blends suppose powerful cell carriers to mold microparticles into hierarchical bone structures. Gelatin, in this case, also increases the Young’s modulus of SF [84].

Collagen–graphene–HA is another candidate for bone repair and regeneration, and drug delivery. The addition of graphene to the scaffold provided it with the capacity to synthesize and fold at the nanometric level [85]. Both graphene and its derivatives have denoted positive outcomes in nanomedicine approaches in bone repair, due to their additional mechanical properties and electrical conductivity, besides atomic structure stability. The search for optimized bone formation and functionalization seems to be an open door, and an area of study that can be deepened with graphene studies [86]. The development of electrospun nanocomposites is another novelty of nanomedicine. The combination of graphene oxide (GO) and nHA with PLA via electrospinning, to create versatile nanofibers, has denoted improvements in synthetic materials, such as PLA in the crystallization process, and biocompatibility. The addition of adequate proportions of nHA and graphene derivatives to PLA obtains a high tensile strength and modulus [87]. The same occurs for scaffolds made of polycaprolactone (PCL)/chitosan/collagen/GO, with the particularity that increased concentrations of GO boost osteogenesis activity, cell attachment, and proliferation [88]. The optimization of these nanofibers with the materials already mentioned keeps being studied. Chitosan, for example, exhibits better opportunities for enhanced proliferation in its carboxymethylated form, testing the PCL/carboxymethyl chitosan composite [89]. Injectable cell-coupled scaffolds, such as osteogenic nanofibrous PCL/collagen, have also demonstrated that technology can mimic the hierarchical architecture of native bone, modulating the sizes of nanofibers in order to provoke osteoblast phenotype progression [90].

Another important and interesting feature of polymer composites is related to their antimicrobial activity, aiding in the prevention of postsurgical infections, or by delivering antibiotics or other agents when the infection is occurring [91]. In this sense, many polymers and polymer composites have been tested, and they have potential antimicrobial activity, in combination with certain antibiotics, to treat osteomyelitis [92]. Chitosan, PLGA, poly(d,l-lactide) (PDLLA), alginate, acrylate gels, and PCL are some of the investigated polymer-based composites that have proven antimicrobial properties against some of the most important bone infectious agents, such as *Staphylococcus aureus* and *methicillin-resistant Staphylococcus aureus (SARM), Staphylococcus epidermidis or Pseudomonas aeruginosa,* amongst others [93,94]. Elastin-like polypeptide–collagen hydrogels are also promising drug carriers, showing both osteoregenerative properties and antibacterial activity [95]. Deepening the antimicrobial properties of polymer composites is a central issue to prevent infections after bone grafting, also representing a potential therapy for established infections, such as osteomyelitis.

Overall, there are some promising results of polymeric composites in the field of bone tissue engineering. However, the main issues to address in the use of polymer composites consist of the enhancement of bone regeneration without causing mechanically induced bone resorption. Thus, achieving proper mechanic characteristics and degradation rates is essential to maximize the use of these constructs as synthetic bone grafts. A detailed review, made by Wagoner Johnson and Herschler [96], claimed that only a few HA/polymer composites overlap the strength of bone, and most are at the upper limit of porosity compared with cancellous bone, but lower than cortical bone. This could be improved by developing original strategies and a combination of different biomaterials in certain proportions to enhance the mechanical properties of the construct, while retaining the bioactivity and biocompatibility of the composite. For instance, Zhao et al. [97] produced 3D porous HA structures with composite coatings based on PDLLA, either alone or in combination with calcium sulfate (CS) and chondroitin sulfate (ChS) powders, and they showed significant improvements in the mechanical properties of the structures. Similarly, Shahi et al. [98] claimed that combining 50% of a β-TCP coating with PHB for 30 s showed desirable properties in bone tissue engineering, according to different morphological and mechanical tests. We encourage this issue to be considered when developing a construct, as it will be of great aid to maximize the characteristics of the composites. Simultaneously, the degradation rates of polymer biomaterials are also key to maximize their use as adequate bone grafts. Furthermore, it is critical to find the balance between the rates of new bone formation and degradation, to promote adequate healing of bone grafts, as shown by Dumas et al. [99]. They observed that augmented PU/allograft composites combined with recombinant BMP-2 altered the normal degradation of the residual PU/allograft (normally between 6 and 12 weeks) to approximate them to a zero-order process (independent of time), increasing the overall bone healing. Barbieri et al. [100] built calcium phosphate with L-lactide/D-lactide copolymers, in various content ratios, using the extrusion method. Interestingly, they described that the higher the amount of HA, the faster the degradation will be, and the stiffness will be increased. However, due to the lower intrinsic viscosity of the polymer phase and in high proportions of HA, they may have lower damping properties and a larger decrease in stiffness, respectively. Importantly, they concluded that by finding an adequate filler content of these constructs, these problems may be diminished. Therefore, it could be concluded that when developing polymer composites, it is critical to focus not only on their degradation time, but also on the osteogenesis/osteodegradation process, and an adequate analysis of the integrators of the construct, the proportion, and the fillers may be of great aid in future studies.

## 5. Polymer Composites and Jawbone Regeneration

**Figure 4:**
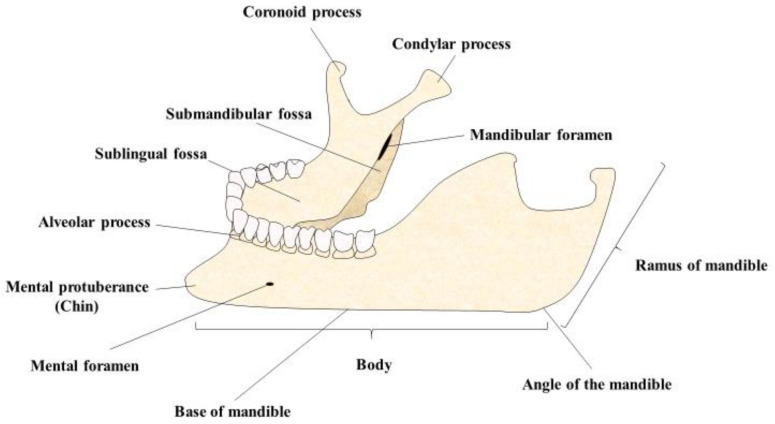
A general picture of the jawbone, where the main anatomical structures are represented.

Jawbones have unique and different regenerative properties in comparison to other bones. For instance, the jaw is a type of irregular bone and most of the studies in this field are conducted in long bones. Hesse et al. [101] found that the bone mineralization density distribution of the jawbone was prominently smaller in comparison to the tibia, supporting the accelerated bone regeneration of the jaw. The last-mentioned property was also due to the proper jaw morphogenesis, and the different process of ossification that occurred in this bone, known as intramembranous instead of endochondral ossification [102]. In addition, due to the proper mastication process, jawbone turnover is frequently conducted in order to prevent bone microdamage. On the other hand, various studies have reported different osteogenic potentials in MSCs derived from the jaw, in comparison to those precedent from long bones [103,104,105]. Similarly, the anatomy of the jawbone (Figure 4) must also be evaluated prior to implantation, as these changes could negatively affect the mandibular vascular supply and the regenerative capacity of the tissue [106]. Overall, these facts must be considered when addressing jawbone regenerative medicine, as this structure presents a unique and particular behavior in comparison to other similar bones. Currently, the use of autogenous bone grafts, either non-vascularized or vascularized, are the gold standard approaches in jawbone regeneration, particularly the fibular free flap, as the fibula is considered the most donatable bone in the human body [107]. However, the use of bone substitutes and tissue engineering are playing a key role in jawbone regeneration, although there is still a long road to discover, as the available evidence is limited, and greater knowledge of the cellular and molecular mechanisms of the mandible is required [108]. Tissue-engineered products that are used in the jawbone may be able to mimic and interact with the native macro and microenvironment of the mandible, adapting to the particular defect of the patient and maintaining the proper functions of this structure. This is achieved through the regeneration triad, which consists of the creation of (1) a biomimetic, bioactive and osteointegrative scaffold that is 3D printed for the jawbone defect, which is frequently combined with a growth factor such as BMP-2; and (2) MSCs [109]. The most important feature of an implant is that it may be directed to not only address the jawbone histology and anatomy, but also the aesthetic and functionality of the mandible [110].

Regarding tissue engineering in the jawbone, cumulative evidence is demonstrating the effectiveness of using different biomaterials, such as natural/synthetic polymers, ceramics, or composites as scaffolds [111]. Frequently, these polymeric composites are combined with MSCs and growth factors. In general, polymer composites might be used either in block (for instance, in chin or the ascending ramus area of the lower jaw) or particulated, adapting better to a concise defect [112]. The alveolar process is part of the jawbone, with many nerves and vessels around this structure, comprising an inner and outer component, containing the tooth sockets on the jawbone, which are in contact with the soft tissue. It is also considered as part of the periodontal tissue, together with the root cementum, periodontal ligament, and the dentogingival junction [113]. In this sense, there are some studies supporting the use of polymer composites to preserve the alveolar process structure, for instance, after a tooth extraction [114]. In this study, Ohba et al. used an HA/collagen composite in the alveolar process in 24 patients, evaluating their results by computed tomography and a bone biopsy. They obtained some promising results from this procedure, clarifying the potential benefits of using this composite in this structure. Simultaneously, polymer composites could be of great aid prior to a dental implant, acting as scaffolds in those cases. Jeong et al. [115] developed a 3D-printed PCL frame and osteoconductive ceramic materials (HA and β-TCP). They observed that these hybrid scaffolds presented high porosity and excellent microstructural interconnectivity, reporting superior results compared to those obtained by the control. Eventually, they concluded that this composite is a promising candidate for minimizing the cost and duration of dental implant surgery. Apart from the composition, other variables must be considered in this field; for instance, previous studies have demonstrated that the rigidity of the composite was an important factor influencing the mobility of the fractured alveolar process, and the use of at least a 0.9 mm wire reduced the fractured alveolar process displacement [116].

On the other hand, Tumedei et al. [117] conducted a retrospective analysis of published papers, from the last 30 years from the Italian Implant Retrieval Center, about the use of barrier membranes for jawbone regeneration. They reported one article that demonstrated the high effectiveness of either resorbable PLA/PGA membranes or expanded polytetrafluoroethylene (e-PTFE) in the treatment of implant dehiscence and fenestrations when associated with autogenous bone chips [118]. Similarly, another study also tested a polymer composite formed by poly(ɛ-caprolactone)-block-poly(oxyethylene)-block-poly(ɛ-caprolactone) and dispersed HA microgranules in vitro. They recommended its uses as periodontal membranes, obtaining an adequate biological tolerance and being virtually resorbable after 6 months [119]. Other lines of research have also evaluated the applications of polymer composites in other jaw regions. Zhu et al. [120] tested a PLGA/MSCs/NELL-1(NEL-like molecule-1) composite in 50 adult goats with an induced 3 × 5 mm osteochondral defect in the mandibular condyle. They observed that, compared to PLGA/MSCs and more prominently with PLGA alone, the composite obtained a complete and rapid restoration of the entire defect after 24 weeks. Another polymer–ceramic composite evaluated in the jawbone was formed by octacalcium phosphate (OCP) and collagen (OCP-collagen). Kawai et al. [121] denoted the potential implications of this composite in a patient with a large mandibular defect, observing complete bone regeneration 12 months after transplantation. Interestingly, they added five-fold OCP-collagen in comparison to previous clinical trials, thereby supporting that finding an adequate amount of the composite is essential to maximize the results. Similarly, a polymer composite of PMMA, PHEMA (poly-hydroxyl-ethyl-methacrylate), and calcium hydroxide (HTR) was proven in mandibular molar type II furcations, reporting similar beneficial outcomes to other graft materials, supporting its use as an adjunctive therapy for these patients [122].

Overall, a growing amount of evidence is supporting the fact that the use of polymer composites might be of great aid in those issues affecting the oral structures, including the periodontal structures, alveolar process, and jawbone. However, future studies should address some issues in this area, including the development of less invasive technologies, the creation of drug carrier and delivery scaffolds, and the establishment of optimized protocols and approaches to achieve complete restoration of craniofacial and oral alterations, in terms of either hard or soft tissues [123].

## 6. Conclusions

**Figure 5:**
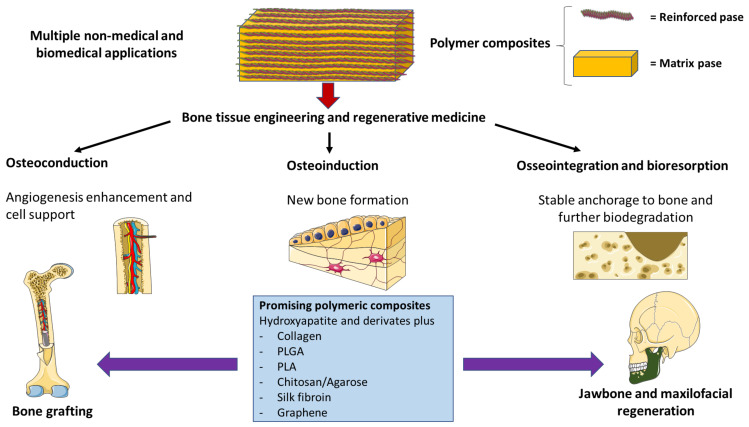
A graphic summary of the ideas transmitted.

Polymer-based composites are promising biomaterials with many biomedical applications, representing an interesting approach in bone tissue engineering (Figure 5). In this paper, we have collected some of the most relevant and updated articles evaluating the role of this composites in bone regenerative medicine, although this field is still in its infancy. Many of the studies are being conducted in long bones, such as tibia or femur, although there are some promising results in other locations, such as the jawbone, alveolar process, and periodontal tissue. HA/collagen, either alone or in combination with other biomaterials, are the best-characterized composites in bone tissue engineering, although there are other biomaterials that should be considered in this area. However, further research and clinical trials are needed, as bone substitutes appear to be a more appropriate approach in bone graft or bone regeneration, particularly when combined with MSCs or different growth factors. The combination of multiple properties of different biomaterials in such innovative ways will be of great aid in advancing this potential area of tissue engineering and biomedicine. 

## Data Availability

Data sharing not applicable.
